# On a Remarkable Case of Ocular Disease Produced by an Electrical Discharge

**Published:** 1849-01

**Authors:** Thomas Cutler

**Affiliations:** Physican to the Dispensaire General at Spa, Belgium


*On a Remarkable case of Ocular Disease produced by an Electrical Discharge.* By Thomas Cutler, M. D., Physican to the Dispensaire General at Spa, Belgium.—I was consulted, a few days since, by Mons. L——, a lieutenant in the Belgian Custom-house service, for a defect of vision, which, at the first moment, I considered to be only a case of advanced amblyopia; but by a more minute examination, I was led to the discovery of the following interesting circumstances:—

Mons. L——, resided at Viel-Salm, on the Prussian frontier, fifty-one years of age, of tall stature, of a sanguineo-nervous temperament, not stout, but of a well-knit frame, and who had been all his life in the enjoyment of robust health, with a remarkably keen vision, was, about six weeks ago, while out on duty, overtaken, in the neighbouring mountains, by a terrific thunder-storm. On the occurrence of a more than usual vivid flash of lightning, followed almost instantaneously by a violent peal of thunder, Mons. L—— felt as if struck by a violent blow on the head, his vision became dim, and he immediately set about returning home. Before reaching his house, the head began to swell to that degree, that upon his arrival he became much alarmed, and sent for the surgeon of the neighbourhood. He describes the swelling to have been equally diffused over the entire scull. He was bled, leeched, several times cupped on the nape of the neck, and otherwise treated antiphlogistically; under which means the tumefaction gradually subsided, and at the end of a fortnight the soft parts returned to their normal condition. The amblyopia continuing, however, to increase, his medical attendant recommended him to visit Spa, and consult me forthwith. I found the pupils much dilated, sluggish in their contraction even before a powerful light, but no change in the transparent humours. Upon closely questioning him, I ascertained that he had become nyctalopic—objects appearing before sunset enveloped in a thick haze, while from that time until morning they were again seen quite distinctly. Suspecting at least a faint central opacity of the lenticular system, I examined him still more attentively by the aid of a *loupe à diaphragme;* but failing to discover any pathological alteration, I subjected him to the well-known test of Purkinje. The result was the same—the refractive structures of the eye were perfectly transparent, and there could be, consequently, no further doubt of its being a true case of nyctalopia.

Willing to ascertain if there were any other phenomena connected with this defect of vision, I tested him upon the perception of colours, and found him anerythroptic. I give you his own appreciation: bright-red he described as pale-yellow; deep-red, deep-yellow; orange, yellow; crimson, the colour of unbleached linen; green, greyish-white or chocolate colour; Vandyk brown, dirty yellow, (jaune bourbeux;) carmine, greyish-yellow; while every diversity of blue he distinguished with ease. Mons. L—— stated that at times, but not frequently, all white objects appeared more or less yellow. I have forgotten to observe, that the green which I exhibited to him upon a white ground, and which he mistook for greyish-white, was nevertheless surrounded by a halo of green.

Seebeck, (see Poggendorff's *Annals*, vol. xlii.,) having accurately tested sixteen anerythroptic individuals, divided them into two classes, those to whom blue, indigo, violet, and green, and those to whom the more refractive rays of red and orange, appeared altered.

How far Brewster's theory for the explanation of anerythropsia, (see Wollaston, *Transactions of the Royal Society*, 1820,) or Herschel's theory, (see "Metropolitan Encyclopedia," § 507, art. on Light,) may find support in this remarkable case, of which the ultimate cause is well attested—namely, electricity,—I leave to others to determine. The facts above recited I cannot but consider worthy of the consideration of physiologists, and natural philosophers, as they furnish at least one undoubted example, that both nyctalopia and anerythropsia may be due to a powerful external cause, while the cause itself may perhaps, in this instance, tend to elucidate the change of structure by which such phenomena can alone take place.

Being, as I have before observed, a healthy man, and unwilling to be placed on the pension list, Mons. L. is anxious to be relieved of his impaired vision, which at present incapacitates him for service. I am therefore treating the case as one of anæsthesia, by the cold douche, and the internal administration of the valerianate of quinine and iron combined with assafœtida. Should this or any subsequent treatment lead to results at all interesting, I shall be happy to communicate them.—*London Lancet.*

